# Plumbagin attenuates traumatic tracheal stenosis in rats and inhibits lung fibroblast proliferation and differentiation via TGF-β1/Smad and Akt/mTOR pathways

**DOI:** 10.1080/21655979.2021.1954580

**Published:** 2021-07-24

**Authors:** Wei Shi, Yuanyuan Fang, Yueming Jiang, Siyang Jiang, Yu Li, Wentao Li, Mingpeng Xu, Michael Aschner, Guangnan Liu

**Affiliations:** a Pulmonary and Critical Care Medicine of The Second Affiliated Hospital of Guangxi Medical University, Nanning, Guangxi, China; b Pulmonary and Critical Care Medicine of the Second People’s Hospital of Nanning, Nanning, Guangxi, China; c Guangxi Medical University, Nanning, Guangxi, China; d Albert Einstein College of Medicine, Bronx, New York, USA

**Keywords:** Tracheal stenosis, plumbagin, tgf-β1, akt, samd2/3, mTOR, fibroblast, proliferation, differentiation

## Abstract

Traumatic tracheal stenosis (TS) is a serious respiratory disease characterized by hyperplasia of airway granulation. Plumbagin (PLB) is a natural naphthoquinone component with anti-fibrotic properties. This research aimed to explore the roles of PLB in alleviating TS and the underlying mechanisms. For *in vitro* studies, lung fibroblasts (IMR-90 cells), with/without PLB treatment or TGF-β1 induction, were used. The viability and proliferation of IMR-90 cells were examined by CCK-8 and EdU incorporation assays. The differentiation of IMR-90 cells was assessed by detecting the mRNA and protein expression levels of collagen (COL)-1 and alpha-smooth muscle actin (α-SMA). Besides, immunofluorescence assay was conducted to evaluate the localization of α-SMA in TGF-β1-induced IMR-90 cells. Moreover, the combination of PLB with/without TβRI (SB-431,542), PI3K/Akt (Ly294002) or mTOR (rapamycin) inhibitor was pretreated on IMR-90 cells after TGF-β1 induction. For *in vivo* studies, a rat model of TS was established. The pathological features and severity of TS were determined by hematoxylin and eosin staining. The protein levels of TGF-β1/Smad and Akt/mTOR pathways were detected for both *in vitro* and *in vivo* models. PLB effectively inhibited the proliferation and differentiation of TGF-β1-induced IMR-90 cells, and suppressed TGF-β1/Smad and Akt/mTOR signaling pathways both *in vivo* and *in vitro*. Furthermore, PLB reduced the degree of TS in rats. Taken together, our results indicate that PLB regulates lung fibroblast activity and attenuates TS in rats by inhibiting TGF-β1/Smad and Akt/mTOR signaling pathways. In conclusion, this study implies that PLB may serve as a promising therapeutic compound for TS.

## GRAPHICAL ABSTRACT

**Figure:**
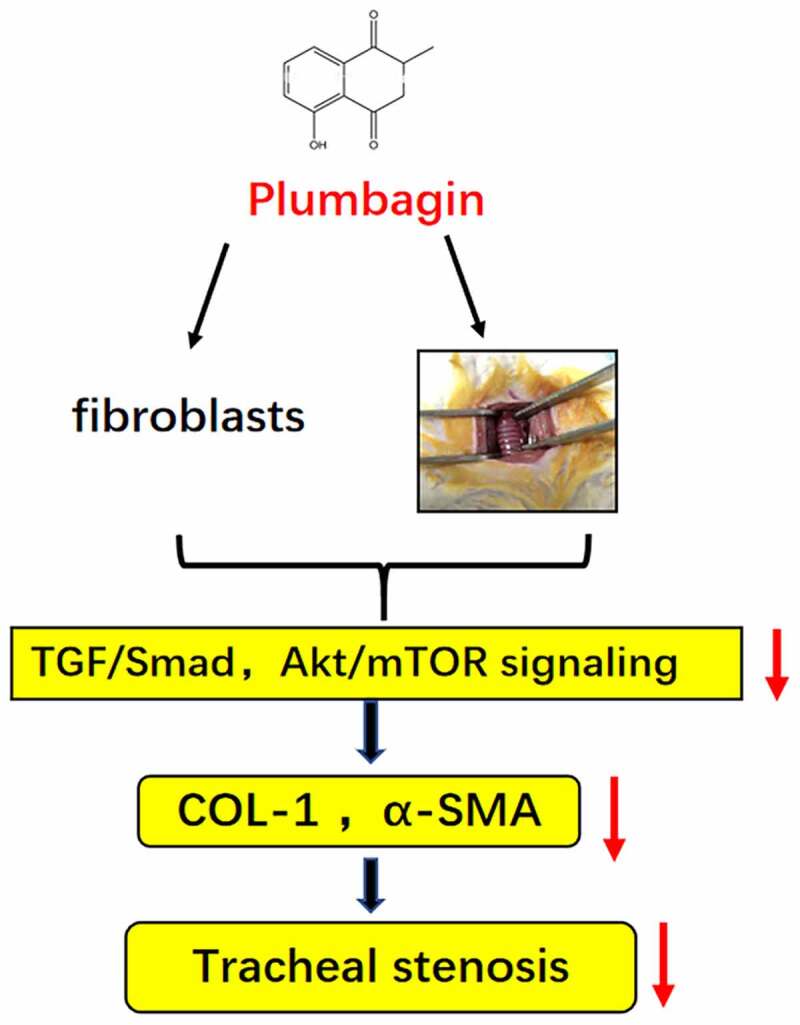

## 1. Introduction

Traumatic tracheal stenosis (TTS, herein after referred to as TS) is commonly caused by hyperplastic granulation and scar formation after tracheal injury, which often manifests as a narrowing of the tracheal lumen [1]. The risk factors for TS include mechanical compression injury after prolonged intubation or tracheotomy, tracheal burns and other injuries. TS may lead to continuous dyspnea or even death (due to asphyxia) in afflicted individuals [2]. The treatment of TS has concentrated mainly on the surgical operation or bronchoscopic procedures [3]. Although invasive treatment can rapidly ameliorate granulation and scarring, not all patients are eligible to undergo invasive interventions [4,5]. In addition, surgically treated patients still face recurrent granulation hyperplasia and restenosis [6]. Therefore, drug therapy is of great value. At present, there is no specific drug therapy for TS. Some anti-inflammatory and anti-fibrosis drugs have moderate efficacy on TS in animal experiments; yet, they are still in the early stages of evaluation [7,8].

After airway injury, tissue repair can lead to the production of fibroblastic cells and collagen-based extracellular matrix proteins, and the ensuing tissue granulation represents a key factor for the development of TS. Microscopic evaluation of the airway stenosis tissue demonstrated the existence of extracellular components such as collagen, fibronectin and proteoglycans. When the fibroblast were stimulated persistently and differentiated into myofibroblast, the symbolic protein α-SMA (alpha smooth muscle actin) could help to promote the strengthening of hyperplastic granulation and once the excessive fibrotic reactions manifested as a ‘bad scar’ [9].

TGF-β belongs to a superfamily of secreted polypeptide growth factors involved in cell proliferation, differentiation, invasion, migration and apoptosis [10,11]. As one of the ligands, TGF-β1 is a prominent molecular regulator of fibrotic process and tissue repair by inducing the proliferation and differentiation of lung fibroblasts [9]. After binding to the TGF-β receptor, it phosphorylates Smad-2 and −3 proteins (or known as the main mediators of TGF signaling) [3,12], transmits extracellular signals to the nucleus to promote collagen synthesis, and integrates with α-SMA to induce myofibroblast formation and granulation tissue production [13]. TGF-β1 can also affect its downstream PI3K/Akt/mTOR1 signaling pathway through non-canonical mechanisms, and activate it secondary to phosphorylation, thus regulating cell proliferation and protein synthesis [11,14,15]. Previous studies have reported that the phosphorylated mTOR1 protein can promote the proliferation of tumor cells and fibroblasts [16], and inhibition of PI3K/Akt/mTOR1 pathway can attenuate lung fibroblast proliferation and alleviate fibrosis [17].

Plumbagin (PLB) is a naturally occurring naphthoquinone substance found in various plants including *Plumbago zeylanica L, Juglans regia, J. cinerea, and J. nigra*. PLB has a variety of biological activities, such as apoptotic induction, anti-inflammatory, anti-fibrotic, anti-tumor and antioxidant effects [18,19]. Several studies on tumor cells have pointed out the ability of PLB to inhibit Akt and mTOR1 pathways, thereby suppressing tumor cell proliferation and differentiation [20,21]. In addition, it has been reported that PLB can regulate the deposition of collagen in wounded skin and promote wound healing in diabetic animal models [22]. More recently, it has also been found that PLB can attenuate the proliferation of lung fibroblasts and inhibits the effects of TGF-β1 on lung fibrosis in mice by downregulating P300 [23]. PLB can also downregulate the overexpression of Smad2 protein caused by lung fibroblasts upon induction with F-828 (a soluble fullerene [C60] derivative) [24]. In a rat liver fibrosis model, PLB has been shown to reduce TGF-β1 expression and exert a protective effect on liver injury accompanied by inflammation and fibrosis [25].

Although TS is hypothesized to be related to abnormal repair after injury, its specific mechanisms have yet to be characterized. In this study, we established a rat tracheal stenosis model to investigate whether PLB attenuated TS through theTGF-β1/Smad and Akt/mTOR pathways. Furthermore, we investigated the suppressive effect of PLB with pathway inhibitors on the fibroblasts activation. To achieve these goals we conducted both *in vivo* and *in vitro* experiments.

## 2. Methods

### 2.1. Cell growth and treatment conditions

Human fetal lung (IMR-90) cells were procured from the Chinese Academy of Sciences' Cell Bank (Shanghai, China), and subsequently cultured in DMEM (Gibco, USA) containing 10% FBS (Gibco, USA) and antibiotics (1% streptomycin and penicillin; SolarBio; Beijing, China). The cells were incubated at 37°C in a humidified atmosphere with 5% CO_2_, and the medium was changed every 2 days. When reaching 60% confluence, cell treatment was carried out. The PLB (purity >98%; MB5765, Meilunbio, China) was prepared at 100 mM as a stock by dissolving in DMSO (Sigma, USA). LY294002 (B-0294, SolarBio; Beijing, China), SB-431,542 (IS1230, SolarBio; Beijing, China), rapamycin (IR0010, SolarBio; Beijing, China) and TGF-β1 (P02279, SolarBio; Beijing, China) were also dissolved in DMSO. All compounds were diluted with culture medium to the final assay concentrations, and DMSO was employed as the control group.

### 2.2. Cell viability assessment

The toxicity of PLB on IMR-90 cells was determined by CCK-8 assay (SG10001, Biosharp, Sichuan, China) in compliance with the manufacturer’s protocol. IMR-90 cells were cultured under three conditions: (1) IMR-90 cells (5,000 cells/well) were grown in 96-well plates for 1 day, and then exposed to 0, 5, 10 and 20 μM of PLB for another 1 day. (2) IMR-90 cells (3,000 cells/well) were cultured with complete medium in 96 well plates for 1 day, induced with 10 ng/mL TGF-β1 for 2 days, and then exposed to 10 μM PLB (in 10% FBS-supplemented DMEM) for 1 day. (3) The cells were pre-treated with 10 μM SB-431,542, 10 μM LY294002 or 100 nM rapamycin for 2 h and induced with TGF-β1 (10 ng/mL) for 2 days, followed by incubation with 10 μM PLB (in 10% FBS-supplemented DMEM) for 1 day. The cells density was the same as in (2). After treatment, the cells were rinsed with PBS, added with 100 mL of CCK-8 solution (10 mL)-containing DMEM per well, and then incubated at 37°C for 1 h. Measurement of optical density (OD) was carried out using a spectrophotometer (Filter Max F3, Molecular Devices, USA) at 450 nm. Cellular viability rate = [(As – Ab)/(Ac – Ab)] x 100%, where As, Ac and Ab are the OD of the experimental group, control group and zero-adjustment group, respectively.

### 2.3. EdU cell proliferation assay

The effect of PLB on TGF-β1-activated IMR-90 cell growth was determined by EdU (5-ethynyl2ʹ-deoxyuridine) incorporation assay. First, IMR-90 cell line was grown into six-well plates, induced with TGF-β1 (10 ng/mL) for 2 days, and then exposed to 10 μM PLB for 1 day. After rinsing in PBS, the cell line was incubated with 1 μM EdU at 37°C for 2 h. Following incubation, the cell line was fixed with 4% paraformaldehyde for 15 min, incubated with 0.5% Triton X-100 for 15 min, and washed 3 times with PBS. Subsequently, the cell line was incubated with a 0.5 mL click additive solution at ambient temperature for 30 min in the dark. Thereafter, the cell line was stained with 1 μg/mL Hoechst 33,342 for 10 min. Lastly, all images were recorded using an Olympus fluorescence microscope (Tokyo, Japan).

The ability of LY294002, SB-431,542 and rapamycin to further inhibit TGF-β1-induced IMR-90 cell proliferation was determined using an analogous EdU Alexa Fluor® 594 proliferation assay (Beyotime, Shanghai, China) in compliance with the manufacturer’s protocol. Briefly, IMR-90 cell line was grown in six-well plates, and then pretreated with 10 μM LY294002, 10 μM SB-431,542 or 100 nM rapamycin for 2 h, induced with TGF-β1 (10 ng/mL) for 2 days, and then exposed to 10 μM PLB (in 10% FBS-supplemented DMEM) for 1 day. Next, the IMR-90 cells were marked, fixed, cleaned and permeated with EdU, followed by the addition of click additive solutions and nuclear staining (all as described above).

### 2.4. Experimental TS model

Sprague–Dawley (SD) rats (male, 280–320 g), specific pathogen free, were procured from Laboratory Animal Center, Guangxi Medical University, Nanning, China. These rats were kept under the control conditions (12:12-h light/dark cycle, temperature: 20 ± 1°C and humidity: 50 ± 10%), and then subjected to 1-week period of acclimatization before experimental testing. Food and distilled water were provided *ad libitum*.

All rats were randomly classified into four groups (n = 6 per group): (1) No template control (NC) group, (2) TS (model) group, (3) TS + normal saline (TS+NS) group, and (4) TS + PLB (TS+PLB) group. The TS model was constructed based on the methods described by Mizokami and coworkers [26]. Tracheotomy was subsequently performed. The subcutaneous tissue was carefully separated with ophthalmic forceps to avoid damage to muscle. When the trachea was exposed completely, an annular tracheal incision was made with a scalpel under the 1–2 rings of the cricoid cartilage. Nylon brush was used to brush the inner wall of the trachea back-and-forth for 8 times from tracheostoma to the distal trachea (about 1 cm length), and then the tracheal incision was sutured in the rats of Group (2), (3) and (4) to establish TS model. In Group (4), the rats were intraperitoneally injected with 4 mg/kg/d PLB for five consecutive days (refer to previous literature) [23,27]. PLB (20 mg) was dissolved in 20 μl DMSO first, then diluted in 50 ml normal saline at a final concentration of 0.4 mg/ml. Meanwhile, the rats in Group (3) were intraperitoneally injected with normal saline at the same volume and days.

After 9 days, the rats were euthanized by intraperitoneally injecting 150 mg/kg (lethal dose) pentobarbital. The specimens were extracted. The narrowing tracheal segment of about 1 cm was cut off for future experiments [28]. Ethical approval for animal experimentation was obtained from the Animal Care and Use Committee at Guangxi Medical University (number: wydw2017-0007). The procedures involving surgery, treatment and postoperative care were performed in accordance with the Guide for the Care and Use of Laboratory Animals (NIH, USA).

### 2.5. Western blot detection

Western blotting was conducted using standard procedures. Total protein was isolated from the treated fibroblasts and tracheal tissues using radioimmuneprecipitation assay buffer (high)-phenylmethylsulfonyl fluoride (RIPA-PMSF; SolarBio, Beijing, China). Subsequently, total protein content was determined by BCA assay kit (SolarBio, Beijing, China). An equal number of proteins were electrophoresed through SDS-PAGE, and then transferred onto nitrocellulose membranes (Merck Millipore, Germany). After inhibiting with 5% skimmed milk for 2 h, the membranes were incubated at 4°C for 12 h with the following primary antibodies: α-SMA (E-AB-34,268; Elabscience, China), COL1 (collagen 1; ab34710), Akt (ABP50629; Abbkine, USA), p-Akt (ab19623), mTOR (ab32028), p-mTOR (ab109268), Smad2 (1:2000, ab40855), p-Smad2 (ab188334), Smad3 (ab40854), p-Smad3 (ab52903), TGF-β1 (ab179695) and β-actin (1:5000, bs-0061 R; Bioss, China). All antibodies were purchased from Abcam (USA), with a dilution of 1:1000, unless otherwise stated. After rinsing 3 times in TBST, the membranes were incubated with the respective secondary antibodies at ambient temperature for 2 h. Lastly, the immunoblots were detected by electrochemiluminescence plus reagent kit (Biosharp, Guangdong, China), and densitometric analysis of each target protein band was conducted using ImageJ software (NIH).

### 2.6. Macroscopic assessment and measurement of TS

The trachea and its surrounding soft tissues were examined macroscopically for TS assessment. The pathological alterations of the airway mucosa and the degree of the lumen stenosis were evaluated by hematoxylin and eosin (H&E) staining. The tracheal tissues were cut at 4-mm thickness and fixed with 10% formalin for over 1 day. After dehydration and clearing, the excised tissues were embedded in paraffin, followed by HE staining. The HE staining was performed as described previously [29]. ImageJ software was employed to observe the area of the lumen at low-power magnification. The degree of stenosis was measured based on the following equation [26]: (1-mucosal surface lumen area/tracheal cartilage lumen area) × 100.

### 2.7. Real-time quantitative PCR

The mRNA levels of COL1 and α-SMA in IMR-90 cells were analyzed in biological triplicates. Total RNA was prepared using Trizol regent (Solarbio, Beijing, China), and then reverse-transcribed with PrimeScript RT Master Mix (Perfect Real Time; BioRad, Hercules, California). Real-time quantitative PCR was conducted on a second-generation PCR instrument (Applied Biosystems®, USA) using TB Green Premix Ex Taq II (Tli RNaseH Plus). The reaction conditions were as follows: 95°C for 30 s, followed by 40 cycles of 95°C for 5 s, 60°C for 30 s and 72°C for 15 s. ACTB was employed as a reference gene for normalizing the gene expression data. Table 1 lists the primer sequences used.

**Table 1.** qPCR primer sequences

Gene	Primer sequences
COL1	Forward: 5ʹ-CGCCCTGGAGCCCCT-3’
	Reverse: 5ʹ-CACCAGCAATACCAGGAGCA-3’
α-SMA	Forward: 5ʹ-CGGGACTAAGACGGGAATCCT-3’
	Reverse: 5ʹ-TACAGAGCCCAGAGCCATTG-3’
ACTB	Forward: 5ʹ-GTCATTCCAAATATGAGATGCGT-3’
	Reverse: 5ʹ-GCTATCACCTCCCCTGTGTG-3’

### 2.8. Immunofluorescence assay

IMR-90 cell line was grown in 24-well plates with glass bottom for 1 day. Once reaching 60–70% confluency, the cells were induced by TGF-β1 (10 ng/mL) for 2 days, except NTC group, and then exposed PLB (0, 5, 10 or 20 μM) for 1 day. After washing 3 times with cold PBS, the cells were fixed with 4% paraformaldehyde at ambient temperature for 15 min, permeabilized with 0.5% Triton-X 100 in PBS for 10 min, and then blocked with 5% bovine serum albumin in PBS. Subsequently, the cell line was incubated with anti-α-SMA antibody (1:100) at 4°C for overnight, and then incubated with the corresponding fluorescent substance-labeled secondary antibody for 1 h. After mounting with DAPI (Solarbio, China) stain, the immunofluorescene images were recorded using an inverted fluorescence microscope with phase contrast (Olympus, Japan).

The paraffin sections were de-waxed and hydrated in the xylene and ethanol, then washed with PBS for 3 times. Citric acid repair solution (0.01 M) was used for antigen repair in boiling water bath for 10 min. After washing three times with PBS, the tissue sections were blocked with 5% BSA at room temperature for 20 min. Next, they were incubated with COL1 antibody (1: 400) overnight at 4°C, then incubated with fluorescent antibody Cy3 (1: 200) at 37°C for 30 min. DAPI was used for counterstaining of the nucleus. After washing with PBS again anti-fluorescence quenching agent, sections were mounted avoiding exposure of the slides to light. Inverted fluorescence microscope and ImageJ software was used for recording and quantitative analysis.

### 2.9. Statistical tests

SPSS V.22.0 (SPSS, Inc., Chicago, USA) was employed for the statistical tests. All results were presented as mean ± standard deviation (SD), and the *in vitro* assays were repeated for 3 times. Statistical difference between groups was compared with one-way ANOVA followed by Newman–Keuls test for normally distributed continuous variables; or otherwise, Kruskal–Wallis test was applied. P < 0.05 was deemed to be statistically significant.

## 3. Results

The purpose of this study was to explore the mechanism of tracheal stenosis and the effect of plumbagin on it. We hypothesized that plumbagin attenuated rat tracheal stenosis after injury by regulating the activation of fibroblast through TGF-β1/Smad and Akt/mTOR signaling pathway. We verified our hypothesis by using the rat model of tracheal stenosis caused by nylon brush injury and fibroblast experiment.

### 3.1. PLB attenuates tracheal stenosis in rats

**Figure 1.**
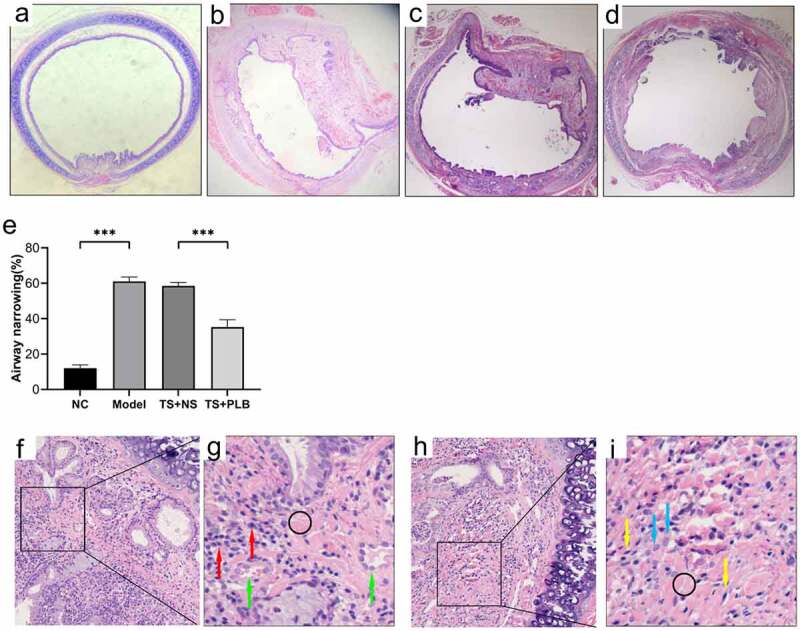
Pathological changes in the airway lumen of the four groups and the stenosis is attenuated by PLB in rat TS model. Upon euthanization, the tracheal tissues were prepared and stained with H&E (a-d,f-i). Representative images of NC (a), model (b), TS+NS (c) and TS+PLB (d) groups. (f-i) is the pathological section of the model group. The magnification of (g,i) is 40 × . (g,i) is a small part of (f,h). Angiogenesis (green arrows) is observed in the submucosal layer with numerous inflammatory cells (red arrows) infiltrated beneath the airway epithelium. The number of fibroblasts (blue arrows) is increased, and fibrocytes (yellow arrows) are dispersed in the thickened submucosa with collagen deposition stained in pink (black circle). The degrees of stenosis are presented in (e).Data represent the mean ± SD of the study groups, n = 6 per group. **P < 0.05, **P < 0.01,*** P < 0.001.The degree of stenosis was measured based on the following equation: (1-mucosal surface lumen area/tracheal cartilage lumen area) × 100

To determine the effect of PLB on tracheal stenosis, we established a rat TS model. H&E staining revealed that hyperplasia of the airway epithelium and thickening of the submucosal layer could induce lumen stenosis. Infiltration of inflammatory cells, increased fibroblasts and fibrocytes in the submucosal layer were also observed. Furthermore, excessive collagen deposition, mucous gland structure and angiogenesis were existed in the thickened submucosa. The thickness of tracheal granulation was markedly higher in TS model rats than in NC rats, implying the successful construction of TS rat model. In addition, the calculated mean percentage of stenosis was remarkably lower in TS+PLB group than in TS+NS group (Figure 1(c-e); P < 0.001), while that of the stenosis group was noticeably higher in the TS model group than in the NC group (Figure 1(a-e); P˂0.001). PLB, intraperitoneally injected at 4 mg/kg/day, effectively attenuated the degree of TS (Figure 1). The mean percentages of TS were 12.04%±1.8%, 61.08%±2.4%, 58.52 ± 1.9% and 35.21%±4.1% in NC rats, model rats, TS+NS rats and TS+PLB rats, respectively. The immunofluorescence expression of COL1 is shown in Figure 6. Compared with control group, the COL1 expression increased significantly in TS model group, and PLB treatment significantly downregulated the COL1 level in TS+PLB rats than in TS+NS group (Figure 6(a-b). P˂0.05)

### 3.2. PLB inhibits TGF-β1/Smad and Akt/mTOR pathways and reduces COL1 and α-SMA expression levels in TS rats

**Figure 2.**
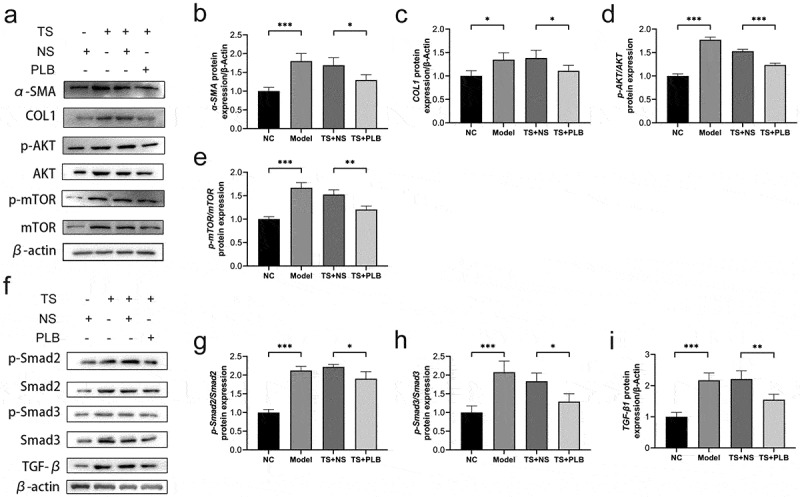
PLB decreases the expression of COL1 and α-SMA as well as suppresses the initiation of Akt/mTOR and TGF-β1/Smad pathways in TS rats. The levels of COL1, α-SMA, p-Akt, Akt, p-mTOR, mTOR, p-Smad2, Smad2, p-Smad3, Smad3 and TGF-β1 proteins were measured by Western blotting and quantified by densitometric analysis (b-e, g-i). Data represent the mean ± SD of the study groups, n = 3 per group. *P < 0.05,**P < 0.01, *** P < 0.001

TGF-β1 plays key roles in regulating fibrosis and scarring, mainly through its downstream Smad pathways. Smad-2 and −3 are two important regulators that contribute to tissue fibrosis via TGF-β1 activation [9]. TGF-β1 can also affect its downstream PI3K/Akt/mTOR1 signaling pathway, which in turn phosphorylates and activates mTOR1 protein to promote fibroblast proliferation [16]. To explore whether PLB can protect against TS injury, the levels of Smad2, Smad3, Akt, TGF-β1 and mTOR proteins were detected. Notably, the protein levels of p-Akt, p-Smad2, p-Smad3, p-mTOR and TGF-β1 were remarkably upregulated in TS rats (Figure 2(d-i) P˂0.01), and PLB treatment markedly downregulated the expression levels of these proteins in TS rats (Figure 2(d-i); P˂0.05). Besides, model group also exhibited a marked increase in the protein levels of COL1 and α-SMA when compared with those in NC (no template control) rats (Figure 2(b-c) P < 0.05, P < 0.001). PLB reduced the protein levels of COL1 and α-SMA in PLB+TS group compared to TS+NS group (P < 0.05). These findings reveal that PLB suppresses the initiation of Akt/mTOR and TGF-β1/Smad pathways and reduces the expression of COL1 and α-SMA *in vivo*.

### 3.3. Toxicity of PLB on IMR-90 cells and inhibitory effects of PLB on TGF-β1-induced IMR-90 cell proliferation

**Figure 3.**
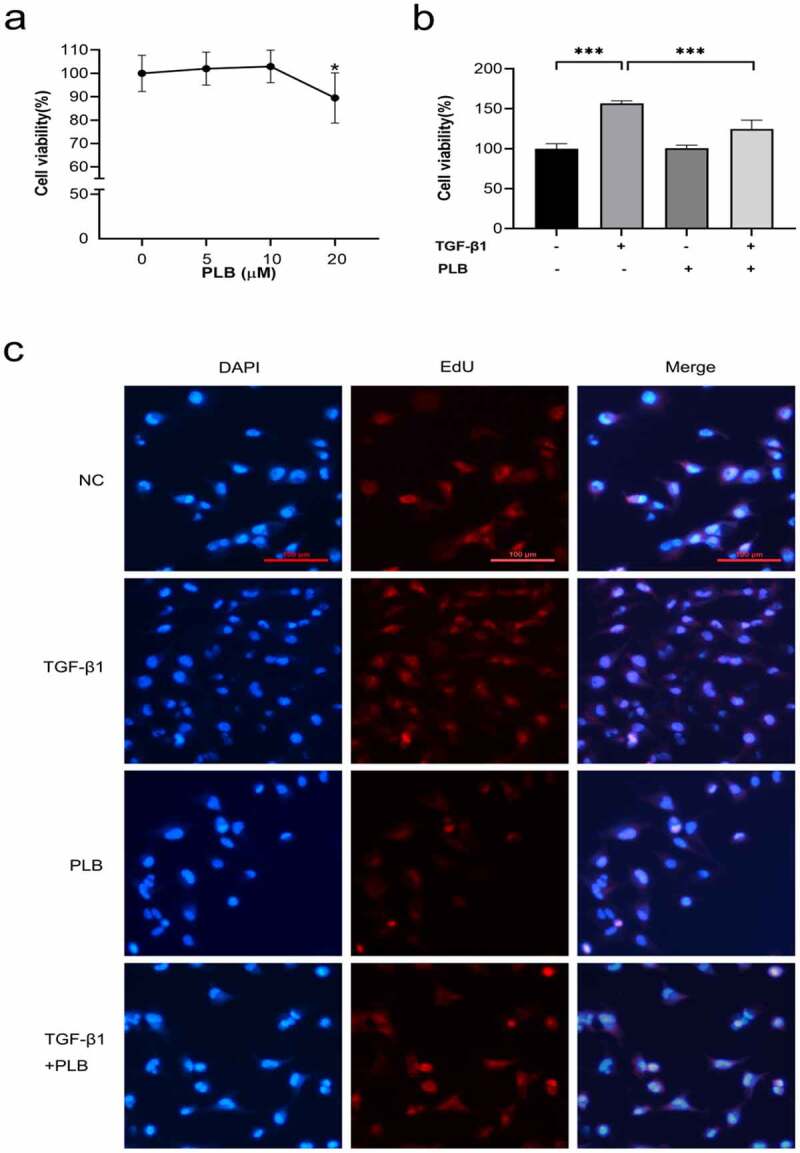
Cytotoxicity of PLB, and its inhibitory effects on TGF-β1-induced IMR-90 cell proliferation. The viability of IMR-90 cells exposed to PLB (0–20 μM, 1 day) was determined with CCK-8 assay (a). The viability of IMR-90 cells exposed to 10 μM PLB for 1 day 10 ng/mL, in the absence or presence of TGF-β1 induction for 2 days, was evaluated by CCK-8 and EdU incorporation assays (b, c). Scale bar = 100 μm. Data represent the mean ± SD of the study groups, n = 6 or 12 per group. *P < 0.05,** P < 0.01,*** P < 0.001

The toxicity of PLB on IMR-90 cells was tested at different concentrations over a time frame of 1 day via cell viability assay (Figure 3a). Given that 20 μM PLB can lead to cytotoxicity after 1 day of incubation (P < 0.05), 10 μM PLB was selected for all subsequent experiments. Next, the inhibitory effects of PLB on fibroblast proliferation after TGF-β1 induction were determined by CCK-8 assay. The results demonstrated that treatment with PLB for 1 day could significantly inhibit the proliferation of 10 ng/ml TGF-β1-induced fibroblasts (Figure 3b; P < 0.001). To further confirm the inhibitory effects of PLB on TGF-β1-induced cell proliferation, EdU incorporation was used, and the results are shown in Figure 3c. It was observed that TGF-β1 induction could remarkably enhance the proliferation of fibroblasts compared with the control groups, while PLB treatment significantly suppressed fibroblast proliferation compared with the untreated control groups.

### 3.4. PLB decreases COL1 and α-SMA expression as well as suppresses Akt/mTOR and TGF-β1/Smad pathways in TGF-β1-induced IMR-90 cells

**Figure 4.**
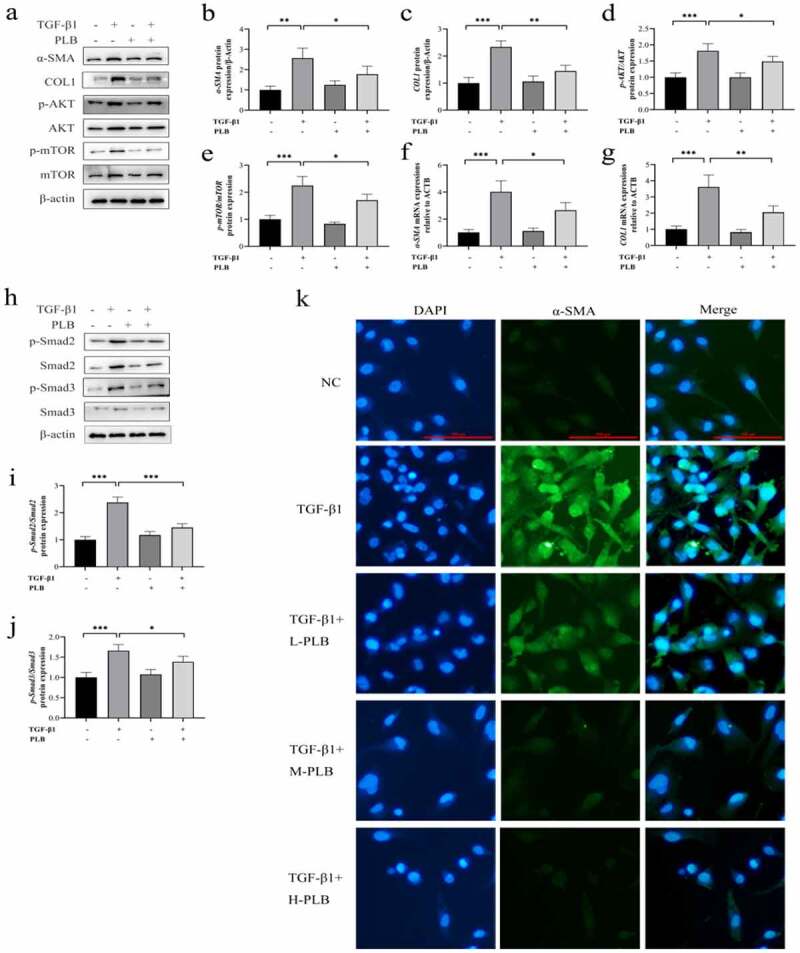
PLB decreases the expression of COL1 and α-SMA as well as suppresses the initiation of Akt/mTOR and TGF-β1/Smad pathways in IMR-90 cells after TGF-β1 induction. the mRNA levels of COL1 (g) and α-SMA (f) were detected by real-time quantitative PCR and normalized with ACTB. The levels of α-SMA, COL1, p-Akt, Akt, p-mTOR, mTOR, p-smad2, smad2, p-smad3 and smad3 proteins were assessed by western blotting and quantified by densitometric analysis (4b-e, 4i-j). (k) Immunocytofluorescence assay of α-SMA. green represents α-SMA-positive cells; and blue stands for DAPI-stained nuclei. scale bar = 100 μm. the concentrations are L-PLB (5 μM),M-PLB (10 μM),H-PLB (20 μM) respectively. The immunocytofluorescence are representative of three independent biological replicates. data represent the mean ± SD of the study groups, n = 3 per group. *P < 0.05,** P < 0.01, ***P < 0.001

The protein (Figure 4b-c) and mRNA (Figure 4f-g) levels of COL1 and α-SMA were remarkably higher in TGF-β1 induction group than untreated control group (P < 0.01, P < 0.001), while PLB treatment downregulated those of COL1 and α-SMA in IMR-90 cells (P < 0.05, P < 0.01). The results of immunofluorescence assay also confirmed that PLB could reverse TGF-β1-induced α-SMA expression (Figure 4k).

To explore whether PLB can promote tissue fibrosis after TS *in vitro*, the levels of Smad2, Smad3, Akt and mTOR proteins were detected in IMR-90 cells after TGF-β1 induction. Noticeably, the protein levels of p-Smad2, p-Smad3, p-Akt and p-mTOR were upregulated in TGF-β1-induced IMR-90 cells (Figure 4(d-e) 4i-j; P˂0.01). However, PLB treatment markedly downregulated the protein levels of p-Smad2, p-Smad3, p-Akt and p-mTOR in TGF-β1-induced IMR-90 cells (Figure 4(d-e) 4i-j; P˂0.05). These findings demonstrate that PLB inhibits TGF-β1/Smad and Akt/mTOR signal transduction *in vitro*.

### 3.5. Effects of PLB on the activity of TGF-β1-induced IMR-90 cells by regulating Akt/mTOR and TGF-β1/Smad pathways

**Figure 5.**
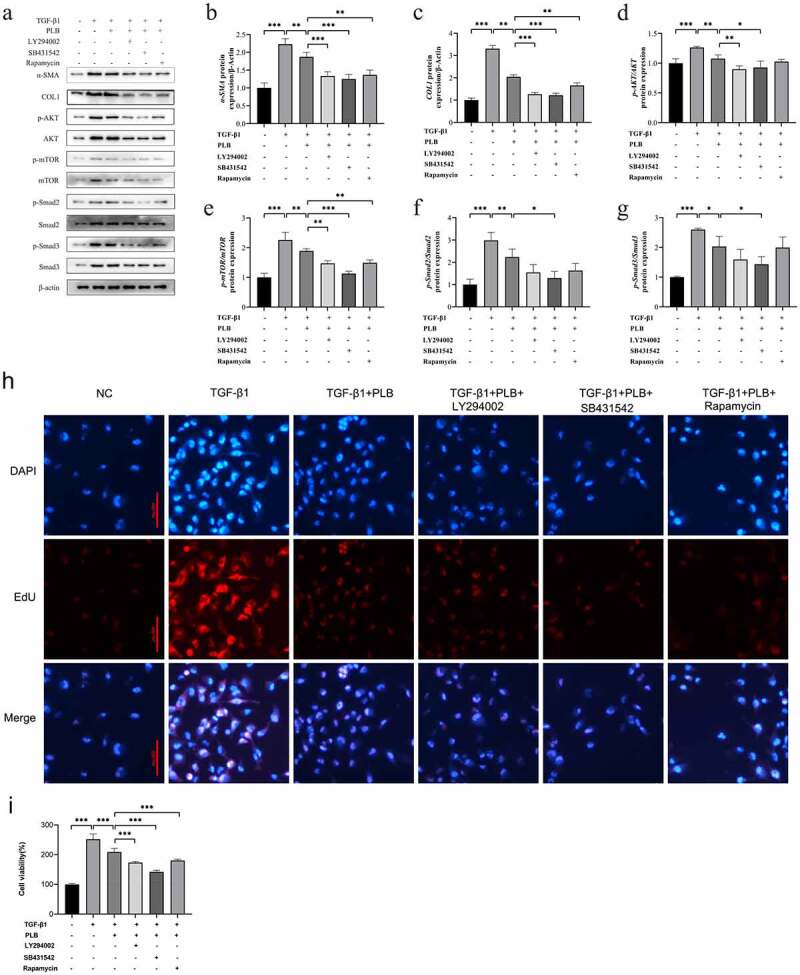
PLB suppresses the activity of TGF-β1-induced IMR-90 cells by regulating Akt/mTOR and TGF-β1/smad pathways. after pretreatment with LY294002, SB-431,542 or rapamycin for 2 h, IMR-90 cells were induced with TGF-β1 for 2 days and subsequently exposed to PLB for 1 day. The levels of p-Akt, Akt, p-mTOR, mTOR, COL1, α-SMA, p-Smad2, Smad2, p-Smad3 and Smad3 proteins in IMR-90 cells exposed to 10 μM LY294002, 10 μM SB-431,542 or 100 nM rapamycin were detected by western blotting (a) and quantified by densitometric analysis (b-g). In the absence or presence of TGF-β1 induction for 2 days, IMR-90 cells were exposed 10 μM PLB for 1 day, followed by 10 μM LY294002, 10 μM SB-431,542 or 100 nM rapamycin for 2 h, respectively, and their viability was measured by CCK8 and EdU incorporation assays (h-i). Scale bar = 100 μm. data represent the mean ± SD of the study groups, n = 6 or 12 per group. *P < 0.05, ** P < 0.01,*** P < 0.001

LY294002, SB-431,542 and rapamycin are the specific inhibitors of PI3K/Akt, TGF-β1/Smad and mTOR signaling pathways, respectively. CCK-8 assay was used to examine whether the combination of PLB with these inhibitors could further affect the viability of TGF-β1-induced cells. As shown in Figure 5i, after pretreatment with 10 μM LY294002, 10 μM SB431,542 or 100 nM rapamycin, the viability of IMR-90 cells was further reduced compared to TGF-β1+ PLB group (Figure 5i; P < 0.001). The results of EdU incorporation assay corroborated these findings (Figure 5h). Collectively, our findings suggest that the combination of PLB with the three inhibitors can decrease cell viability and proliferation by inhibiting Akt/mTOR and TGF-β1/Smad pathways.

Western blot assay was conducted to assess whether the combination of PLB with the three inhibitors could further affect the protein expression of the target pathways in TGF-β1-induced cells. As shown in Figure 5b-g, PLB downregulated the protein levels of p-Akt, p-Smad2, p-Smad3 and p-mTOR in TGF-β1-induced IMR-90 cells (P < 0.05, P < 0.01); LY294002 plus PLB further decreased those of COL1, α-SMA, p-Akt and p-mTOR (P < 0.01, P < 0.001); SB-431,542 plus PLB further decline those of COL1, α-SMA, p-Akt, p-Smad2, p-Smad3 and p-mTOR (P < 0.05, P < 0.001); and rapamycin plus PLB further reduced those of COL1, α-SMA and p-mTOR to varying degrees (P < 0.01), when compared to PLB alone. Besides, as shown in Figure 5(b-c), LY294002, SB-431,542 and rapamycin further enhanced the protective effects of PLB on tissue fibrosis and counteracted TGF-β1-induced fibrotic features by decreasing the levels of COL1 and α-SMA in TGF-β1 and PLB co-treated IMR-90 cells (P < 0.01, P < 0.001). Overall, these findings indicate that PLB attenuates TGF-β1-induced fibrogenesis by suppressing TGF-β1/Smad and Akt/mTOR pathways in IMR-90 cells after TGF-β1 induction.

## 4. Discussion

In this study, we found that PLB attenuated TS induced by mechanical injury in rats. After pretreatment with LY294002, SB-431542 or rapamycin, PLB led to a greater inhibition of TGF-β1-induced IMR-90 cell proliferation and differentiation via TGF-β1/Smad and Akt/mTOR pathways. These results highlight the mechanisms underlying the efficacy of PLB in alleviating TS.

**Figure:**
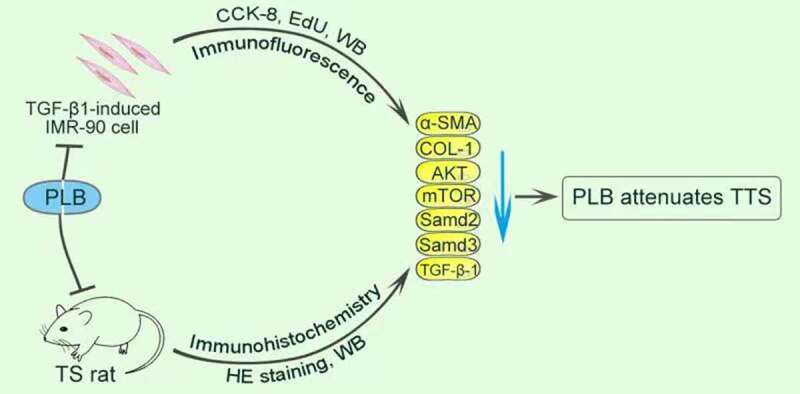

TS is mainly characterized by fibroblast differentiation and proliferation, together with an excessive production of collagen after tissue repair [30]. The repair process after tissue injury can be divided into three stages: the initial stage is characterized by an inflammatory reaction; the middle stage begins with hyperplastic granulation due to fibroblast differentiation and proliferation, collagen deposition and hyperplasia; and the last stage is characterized by tissue remodeling, where fibroblasts secrete matrix metalloproteinases and fortify granulation [31]. Taking advantage of previously published methods for experimental TS [26,28], a rat TS model was successfully established. H&E staining revealed inflammatory cell infiltration, excessive collagen deposition, mucous gland structure, angiogenesis, and increased fibroblasts and fibrocytes in the submucosal layer. Furthermore, irregular thickening and narrowing of the inner wall of the lumen were apparently observed.

**Figure 6.**
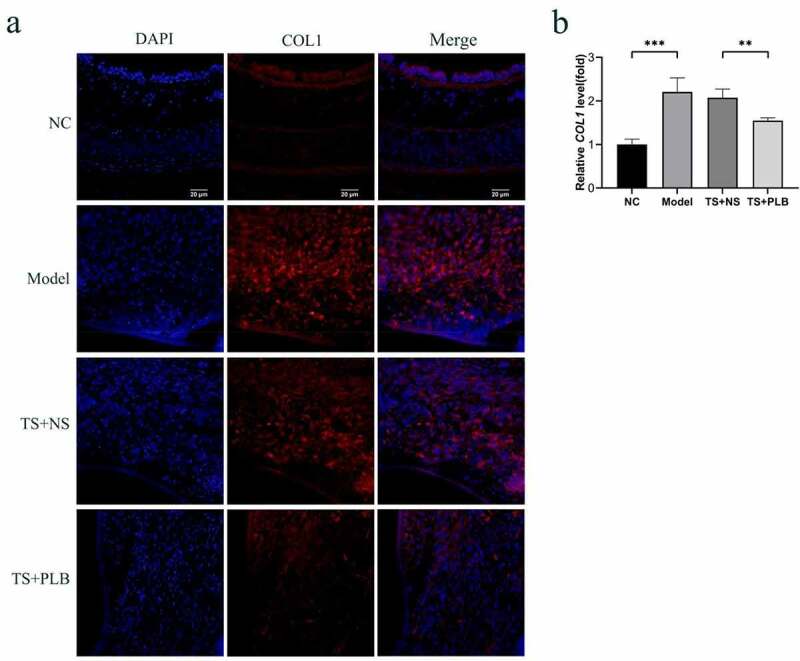
The expression of COL1 in the different groups determined by immunofluorescence (a,b). Scale bar = 20 μm. Red fluorescence ((Cy3, red staining) for the detection of the target protein COL1, blue fluorescence (DAPI staining) for the nucleus.Data represent the mean ± SD of the study groups, n = 6 per group.*P < 0.05,**P < 0.01,***P < 0.001

PLB has a pleiotropic effect on various cellular functions. Some studies have reported that PLB displays anti-fibrotic properties in the lung, kidney and liver [23,25,32]. In this study, our data showed that PLB regulated tissue repair and collagen deposition in TS rats. Immunofluorescence assay showed that the level of COL1 was decreased significantly in TS+PLB group than in TS+NS group (Figure 6a-b). Moreover, PLB inhibited the proliferation and differentiation of TGF-β1-induced IMR-90 cells, as demonstrated by the CCK-8 and EdU methods (Figure 3c, Figure 4k), and the decreased expression of COL1 and α-SMA. However, these effects were not apparent in the absence of TGF-β1 induction, suggesting that PLB only regulates active fibroblasts after tissue injury. Furthermore, the immunocytofluorescence results of α-SMA in TGF-β1-induced IMR-90 cells showed that the increased concentrations of PLB could lead to a decrease in α-SMA levels. *In vivo*, PLB attenuated the narrowing of the lumen and collagen deposition (Figure 1a-c). The results of Western blot analysis also indicated that COL1 and α-SMA were decreased after PLB treatment *in vivo*. Altogether, our findings reveal that PLB can affect the repair of damaged tissues by regulating lung fibroblast proliferation and differentiation.

TGF-β1 is an important regulator of tissue fibrosis. Its canonical pathway TGF-β1/Smad induces the production of myofibroblasts and expresses the characteristic protein of myofibroblasts (e.g., α-SMA) [9]. By phosphorylating the downstream pathway, the activated Smad2 and Smad3 proteins can translocate into the nucleus to promote pro-fibrosis gene transcription. It has been found that TGF-β1 is overexpressed in benign airway stenosis [12,33]. The levels of Smad2 and Smad3 have also been shown to increase in human airway granulation fibroblasts *in vitro* [34]. In the present study, TGF-β1 protein levels were elevated in TS rats, and this effect was attenuated by PLB. Interestingly, p-Smad2/3 proteins also increased in TS rats compared to untreated rats (P˂0.05), and PLB decreased p-Smad2/3 proteins in both TS rat model and TGF-β1-induced IMR-90 cells. Previous research has shown that TGF-β1 can affect organ fibrosis through the noncanonical PI3K/Akt/mTOR pathway [35], and recent studies have demonstrated that this pathway is associated with the regulation of lung fibroblast proliferation [3,35]. mTOR protein expression can be regulated by several signals, such as growth factor, nutrition, energy, etc. mTOR is also a classic molecular factor that plays crucial roles in regulating cell proliferation and protein synthesis [3,36]. Several studies have indicated that mTOR may be independent of PI3K signal and be solely regulated by TGF-β1 to affect collagen formation [37]. In pulmonary fibrosis, the inhibition of mTOR with rapamycin alone has been shown to reduce collagen formation [35]. Namba et al. [38] used rapamycin to treat fibroblasts isolated from the biopsies of patients with laryngo-TS, and found that the anti-fibroblast effect of this drug was characterized by the reduced proliferation of human laryngo-TS fibroblast *in vitr*o. Xiao et al. [3] demonstrated the protein level of mTOR was increased in rabbit TS tissue. Similarly, our data showed that the protein levels of Akt, p-Akt, mTOR and p-mTOR were increased both *in vivo* and *in vitro*; however, these proteins were decreased significantly by PLB. Collectively, our study implies that PLB attenuates TGF-β1/Smad and Akt/mTOR pathways in TS rat model and TGF-β1-induced IMR-90 cells.

To further decipher the mechanism by which PLB ameliorates wound healing via TGF-β1/Smad and Akt/mTOR signaling, we selected TβRI inhibitor (SB431542), PI3K/Akt inhibitor (LY294002) and mTOR inhibitor (rapamycin) to pretreat in TGF-β1-induced IMR-90 cells. It was observed that the protein level of p-Akt was further decreased by PLB plus SB431542 or LY294002 (Figure 5d), that of p-mTOR was further decreased by PLB plus SB431542, LY294002 or rapamycin (Figure 5e), and that of p-Smad2/3 was further decreased by PLB plus SB431542 (Figure 5f). Upon induction of TGF-β1 and pretreatment with these three inhibitors, PLB resulted in a marked reduction of α-SMA and COL1 in IMR-90 cells (Figure 5b-c). The proliferation of IMR-90 cells was also further attenuated after pretreatment with SB431542, LY294002 and rapamycin, as revealed by CCK-8 and Edu incorporation assays (Figure 5h-I; P < 0.001). These results strongly suggest that TGF-β1/Smad and Akt/mTOR pathways are associated with the protective effects of PLB on TGF-β1-induced lung fibroblast differentiation and proliferation.

## 5. Conclusion

In summary, our results suggest that TGF-β1/Smad and Akt/mTOR pathways are activated during the occurrence of traumatic-induced TS. Interestingly, PLB can regulate TGF-β1/Smad and Akt/mTOR pathways to inhibit the activity of TGF-β1-induced IMR-90 cells, which also reflect a mechanism by which PLB attenuates TS in rats. This study indicates that PLB may serve as a novel therapeutic drug to reduce the degree of TS.

## Data Availability

All data generated or analyzed during this study are included in this published article.

## RESEARCH HIGHLIGHTS

1. The expression levels of p-Akt, p-Smad2, p-Smad3 and p-mTOR are increased in rat tracheal stenosis
2. Plumbagin attenuates rat traumatic tracheal stenosis
3. Plumbagin suppresses lung fibroblast proliferation through TGF-β1/Smad and Akt/mTOR signaling

Plumbagin can attenuate rat tracheal stenosis via TGF-β1/Smad and Akt/mTOR signaling.
